# The Ankyrin Repeats and DHHC S-acyl Transferase Domain of AKR1 Act Independently to Regulate Switching from Vegetative to Mating States in Yeast

**DOI:** 10.1371/journal.pone.0028799

**Published:** 2011-12-08

**Authors:** Piers A. Hemsley, Claire S. Grierson

**Affiliations:** School of Biological Sciences, University of Bristol, Bristol, United Kingdom; Purdue University, United States of America

## Abstract

Signal transduction from G-protein coupled receptors to MAPK cascades through heterotrimeric G-proteins has been described for many eukaryotic systems. One of the best-characterised examples is the yeast pheromone response pathway, which is negatively regulated by AKR1. AKR1-like proteins are present in all eukaryotes and contain a DHHC domain and six ankyrin repeats. Whilst the DHHC domain dependant S-acyl transferase (palmitoyl transferase) function of AKR1 is well documented it is not known whether the ankyrin repeats are also required for this activity. Here we show that the ankyrin repeats of AKR1 are required for full suppression of the yeast pheromone response pathway, by sequestration of the Gβγ dimer, and act independently of AKR1 S-acylation function. Importantly, the functions provided by the AKR1 ankyrin repeats and DHHC domain are not required on the same molecule to fully restore WT phenotypes and function. We also show that AKR1 molecules are S-acylated at locations other than the DHHC cysteine, increasing the abundance of AKR1 in the cell. Our results have important consequences for studies of AKR1 function, including recent attempts to characterise S-acylation enzymology and kinetics. Proteins similar to AKR1 are found in all eukaryotes and our results have broad implications for future work on these proteins and the control of switching between Gβγ regulated pathways.

## Introduction

Heterotrimeric G-protein signalling pathways are found throughout eukaryotes and are involved in a wide range of signal transduction events. Heterotrimeric G-proteins, comprising Gα, Gβ and Gγ subunits, are activated by ligand bound G-protein coupled receptors (GPCRs). This generally leads to dissociation of Gα from the Gβγ dimer, both of which are involved in distinct signalling activities. Little is known about which proteins link signalling from Gβγ dimers to downstream effectors.

The mating pheromone response of *Saccharomyces cerevisiae* is one of the best characterised GPCR pathways. Many proteins affecting responses to mating pheromone have been identified and characterised resulting in a broad knowledge base that is useful for further dissection of GPCR pathways. AKR1 has long been known to be a negative regulator of the mating pathway in *S. cerevisiae* yet the mode of suppression is unknown. The gross phenotypic defects of *akr1Δ* mutants have been proposed to be a result of simultaneous activation of both vegetative and mating pathways [1]. *akr1Δ* cells are defective for endocytosis of the a-pheromone GPCR STE3 [2] as a result of YCK2 mis-localisation [3] and show up-regulation of the STE20/STE11/STE7/FUS3 MAPK mating pathway due to increased Gβγ activity [1] leading to partial cell cycle arrest and activation of mating pathway morphogenesis genes in the absence of mating pheromone. These defects result in an abnormal phenotype during vegetative growth where cells frequently form multiple buds, have over-elongated buds and often fail to complete cytokinesis resulting in a multi-nucleate cell mass with pseudohyphal characteristics. *akr1Δ* cells are not however impaired in mating efficiency or up regulation of the mating pathway in response to pheromone [1], [4]. These data demonstrate that AKR1 is responsible for suppressing basal Gβγ mediated mating pathway signalling in the absence of mating pheromone, but does not prevent full mating pathway activation once pheromone is detected [4].

AKR1 has more recently been demonstrated to be a Protein S-acyl transferase (PAT), also known as a palmitoyl transferase, with a diverse range of substrates including the casein kinases YCK1 and 2 [5], LCB4 [6], MEH1, SNA4, LSB6 as well as 3 proteins of unknown function [7]. AKR1 is responsible for the addition of lipid groups through thioester linkages (S-acylation) to promote or increase the membrane association of the target protein [5]. YCK2 is anchored to the membrane by two S-acyl groups where it aids in many cellular processes, including septin organisation to maintain correct cell shape [8], [9], [10], and phosphorylates both STE3 [3] and the FUR4 uracil permease [11] to promote their endocytosis. The defects in YCK2 S-acylation and STE3 endocytosis are thought to be major contributors to the *akr1Δ* phenotype [5], [10].

AKR1 is an integral membrane protein and contains 6 ankyrin repeats and a DHHC PAT domain [12], although in AKR1 the core DHHC motif is changed to DHYC [5]. Proteins with the same domain architecture are found across all eukaryotes, with typically one to two per genome. DHHC domain containing proteins have been shown to be responsible for S-acyl transferase activity across eukaryotes [5], [13], [14], [15], [16], [17]. It is not known however if, and how, the ankyrin repeats of the ankyrin repeat containing subset of PATs contribute to S-acylation. Not all PATs contain ankyrin repeats, indicating that ankyrin repeats may not be required for all S-acylation events.

Using functional complementation assays of *akr1Δ* defects, *in-vivo* S-acylation assays and protein-protein interaction assays we show that both the DHHC PAT domain and the ankyrin repeats of AKR1 independently contribute to the regulation of GPCR/Gβγ signalling. Our results also show that AKR1 molecules regulate each other's S-acylation at sites other than the DHYC cysteine and this affects the levels of AKR1 protein in the cell. The ankyrin repeats promote AKR1 mediated S-acylation of YCK2, but also affect Gβγ signalling by a separate route that does not involve AKR1 S-acylation activity. Our results also have important implications for studies of AKR1 and PAT function, including recent attempts to characterise the enzymology and kinetics of S-acylation.

## Results

### AKR1 molecules increase each other's S-acylation at site(s) other than the DHHC cysteine by a mechanism that proceeds without the AKR1 ankyrin repeats and affects the stability of the AKR1 protein

**Figure 1:**
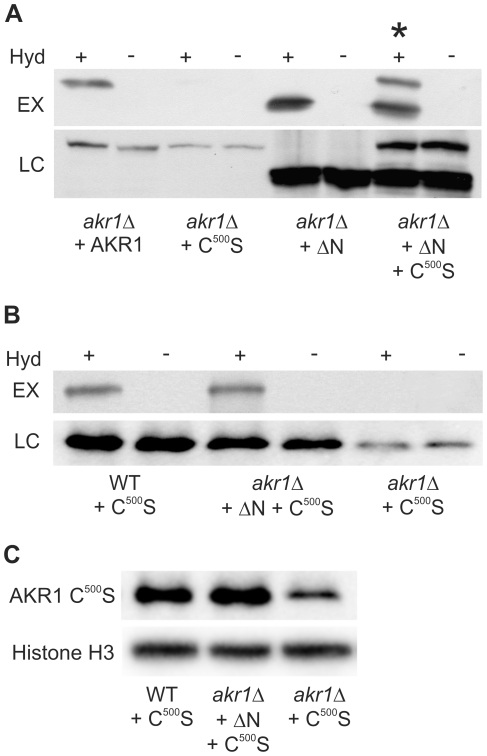
AKR1 S-acylates itself *in trans* and this does not require the ankyrin repeats. S-acylation assays of AKR1 variants expressed in yeast were performed using the biotin switch method whereby hydroxylamine is used to specifically cleave S-acyl groups revealing sulfhydryls which are subsequently labelled with biotin. Samples are then passed through a neutravidin column and S-acylation state is assayed as a function of recovery by the column using antibodies against the protein of interest. Negative controls substitute Tris, which does not cleave S-acyl groups, for Hydroxylamine. +indicates presence of hydroxylamine, −indicates absence of hydroxylamine. EX –S-acylated AKR1 variants detected by the biotin switch method, LC – loading control to show the total amount of AKR1 variant (regardless of S-acylation state) in each sample. (**A**) AKR1 is able to covalently bind acyl groups (auto-S-acylate) and disruption of the DHHC domain by introduction of the C^500^S mutation abolishes this auto acylation. AKR1 ΔN maintains the ability to auto acylate and co-expression of AKR1 ΔN and AKR1 C^500^S leads to S-acylation of AKR1 C^500^S (upper band in lane marked with *). (**B**) S-acylation of AKR1 C^500^S *in trans* occurs in a wild type background. Expression of AKR1 C^500^S in WT cells (AKR1 fully functional) produces the same S-acylation of AKR1 C^500^S *in trans* as co-expression of AKR1 C^500^S with AKR1 ΔN in *akr1Δ* cells. (**C**) Western blot showing that the total amount of AKR1 C^500^S detected increases in conditions where this protein is S-acylated *in trans*, suggesting that S-acylation promotes AKR1 stability. Expression of AKR1 C^500^S in WT cells or co-expression in *akr1Δ* cells with AKR1 ΔN leads to higher levels of AKR1 C^500^S being detected than expression of AKR1 C^500^S alone in *akr1Δ* cells. LC – loading control: Histone H3.

AKR1 encodes a DHHC Protein S-Acyl Transferase (PAT) and published evidence suggests that PATs auto-S-acylate to form a stable catalytic intermediate through a thioester bond [5], [18]. The majority of PATs, unlike AKR1, do not contain ankyrin repeats, suggesting that ankyrin repeats are not essential for S-acylation activity. We deleted the ankyrin repeats of AKR1 to produce AKR1 ΔN (Figure S1) and show that this truncated protein is still S-acylated when expressed in-vivo, suggesting that it retains the ability to auto-S-acylate (Figure 1A). These results indicate that AKR1 ΔN may be able to function as a PAT and the ankyrin repeats are not essential for S-acyl group binding.

Several previous reports have suggested that the DHHC cysteine of AKR1 is required for formation of a stably S-acylated catalytic form, and this residue is believed to be essential for PAT activity [5], [16], [19]. AKR1 C^500^S, in which this cysteine is mutated (Figure S1), does not form an S-acylated intermediate or act as a PAT (Figure 1A) [5]. Figure 1A shows that although AKR1 C^500^S is not S-acylated when expressed in *akr1Δ* mutants [5] it is S-acylated when co-expressed with AKR1 ΔN. These results show that AKR1 molecules that do not have a DHHC cysteine can be S-acylated, demonstrating that S-acylation of AKR1 can occur at sites other than the DHHC cysteine. They also suggest that AKR1 molecules S-acylate each other *in trans* and demonstrates that AKR1 ΔN is able to act as a PAT *in-vivo*. These results are surprising because AKR1 monomers do not form a complex detectable by either co-immunoprecipitation [12] or yeast-2-hybrid assays designed for membrane proteins (Figure S2), although negative effects on higher order structures in these assays may have prevented detection of interactions, and cannot be ruled out. To establish that our observations are not a peculiarity of the AKR1 ΔN background we expressed epitope tagged AKR1 C^500^S in the WT background and again observed S-acylation of AKR1 C^500^S (Figure 1B).

AKR1 C^500^S is detected at lower levels by western blot when expressed in *akr1Δ* cells, where it is not S-acylated, compared to WT cells or *akr1Δ* cells co-expressing AKR1 ΔN, in which AKR1 C^500^S is S-acylated (Figure 1C). These results suggest a role for S-acylation of AKR1 outside of the DHHC motif in maintaining the stability of the AKR1 protein, and indicate that *akr1Δ* mutants expressing AKR1 C^500^S alone should be thought of as not only defective for S-acylation activity [5], but also as having reduced levels of AKR1 protein and therefore the ankyrin repeats.

### The DHHC domain of AKR1 S-acylates YCK2 in the absence of the ankyrin repeats

**Figure 2:**
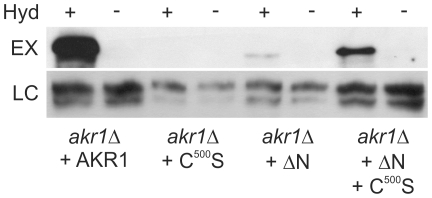
S-acylation of over expressed GFP-YCK2 requires the DHHC domain of AKR1. S-acylation of GFP-YCK2 is supported by AKR1 or AKR1 ΔN but not by AKR1 C^500^S. The presence of the AKR1 ankyrin repeats on the non-S-acyl transferase competent AKR1 C^500^S improves S-acylation of GFP-YCK2 when co-expressed with AKR1 ΔN. S-acylation states were assayed by the biotin switch method, +indicates presence of hydroxylamine, −indicates absence of hydroxylamine. EX – S-acylation state of GFP-YCK2, LC – loading control for GFP-YCK2.

Yeast lacking AKR1 are misshapen, with cells frequently forming multiple or branched buds and failing to complete cytokinesis [1], [4]. These *akr1Δ* phenotypes are likely due to mis-localisation of the type-I casein kinases YCK1 and 2 [5], [10]. YCK2 is thought to phosphorylate STE3 at the plasma membrane leading to STE3 endocytosis and reducing basal Gβγ signalling thereby contributing to mating pathway suppression in the absence of pheromone. YCK2 is reliant upon dual S-acylation by AKR1 for localisation to the plasma membrane and YCK2 is not S-acylated and is mislocalised in *akr1*Δ cells and *akr1*Δ cells expressing AKR1 C^500^S [5], [10] indicating a specific role for the DHHC domain in S-acylation of YCK2. Figure 2 shows that AKR1 ΔN is capable of acting as a PAT, but cells expressing AKR1 ΔN S-acylate less over expressed GFP-YCK2 *in-vivo* than cells expressing full-length AKR1 (approximately 3.2–5.6 fold depending on the experiment, data standardized to input levels of GFP-YCK2). Co-expression of AKR1 C^500^S and AKR1 ΔN promotes S-acylation of over expressed YCK2 to a significantly greater extent (approximately 2.1–3.6 fold) than AKR1 ΔN alone but still less than full length AKR1 (Figure 2). The reduction in AKR1ΔN S-acylation efficiency towards GFP-YCK2 may be due to the effects of overexpression of GFP-YCK2 from the GAL1 promoter as AKR1ΔN is able to S-acylate AKR C^500^S expressed from it's native promoter to the same level as WT (Figure 1A, 1B). Loss of the ankyrin repeats may also lead to a failure to form a full or stable YCK2 S-acylation complex. Preliminary phenotypic analyses of AKR1 and YCK2 dependant phenotypes for an AKR1 construct lacking the ankyrin repeats and carrying the C^500^S mutation were identical to *akr1Δ* null mutants indicating that these are the only two functionalities of AKR1 affecting our observations (data not shown). We therefore decided that assaying this construct for its ability to S-acylate YCK2 was not required. These data confirm that the DHHC cysteine is required for YCK2 S-acylation and demonstrate that the ankyrin repeats are not an absolute requirement. However the ankyrin repeats do appear to increase YCK2 S-acylation by an unknown mechanism that does not require the ankyrin repeats and DHHC domain to be on the same molecule.

### The ankyrin repeats of AKR1 interact directly with Gβγ and are required to suppress Gβ activity in STE4 over expression assays

Data in Figure 2 indicates that the ankyrin repeats of AKR1 are not an absolute requirement for AKR1 PAT activity but these data do not suggest an alternative role for them. AKR1 has been shown to negatively regulate the effect of Gβγ on the mating pathway in the absence of mating pheromone [1], [4]. AKR1 also interacts with STE18/Gγin yeast-2-hybrid assays [1]. Published evidence does not support the hypothesis that AKR1 regulates Gβγ by S-acylation because, although Gβγ requires S-acylation of the STE18 Gγ subunit for localisation to the plasma membrane [20], [21], previously published data demonstrated that two other DHHC S-acyl transferases, ERF2 and SWF1, are responsible not AKR1 [7].

**Figure 3:**
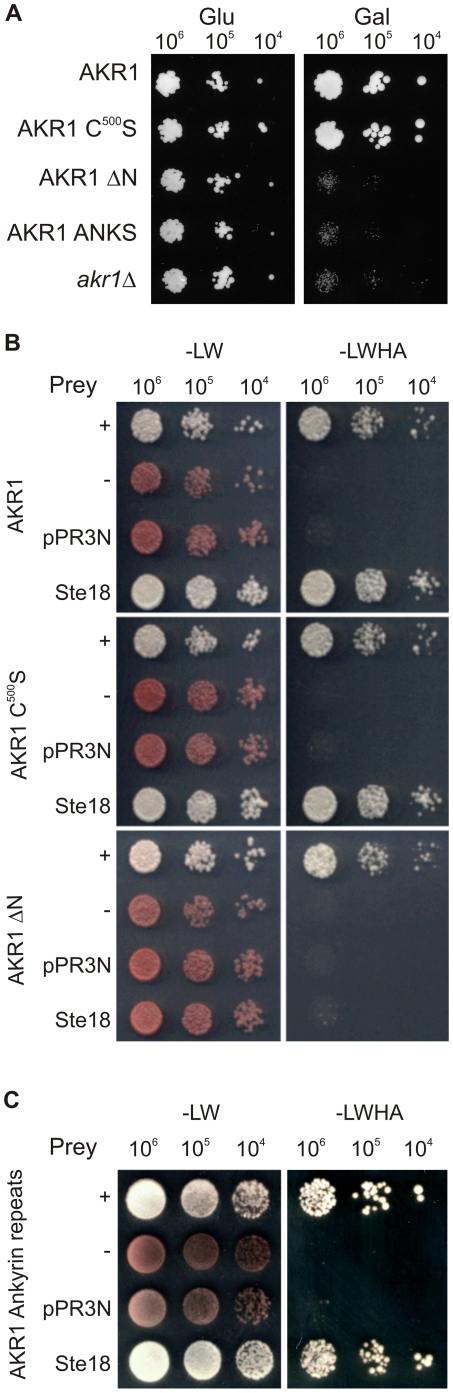
AKR1 interacts with Gβγ through its ankyrin repeats. (**A**) Over-expression of the Gβ subunit STE4 from the GAL1 promoter of pJB9 leads to cell cycle arrest and failure to grow on galactose medium (Gal). All strains grew well on glucose medium (Glu). Over expression of AKR1 or AKR1 C^500^S from high copy number vectors suppresses STE4 over expression induced cell cycle arrest whereas AKR1 ΔN or non-membrane associated AKR1 ankyrin repeats (AKR1 ANKS) do not. (**B**) Yeast-2-hybrid analysis of the interaction between AKR1 variants and the Gγ subunit STE18. AKR1 and AKR1 C^500^S both suppressed the *ade2Δ* red phenotype and produced strong growth on medium selecting for activation of *HIS3* and *ADE2* reporter genes as a result of interaction with STE18 whereas AKR1 ΔN did not. –LW: control medium, -LWHA: selection medium for reporter activation. +denotes pAI (positive control vector), −denotes pDL2 (negative control vector), pPR3N – empty prey vector control, STE18 – pPR3N expressing STE18. (**C**) Yeast-2-hybrid assay demonstrating direct interaction between the ankyrin repeats of AKR1 and the Gγ subunit STE18. The AKR1 ankyrin repeats suppress the *ade2Δ* red phenotype and produce strong growth on medium selecting for activation of *HIS3* and *ADE2* reporter genes as a result of the AKR1 ankyrin repeats interacting with STE18. Controls are the same as for part B.

Over expression of the Gβγ dimer β subunit STE4 leads to cell cycle arrest but high levels of AKR1 expression prevent this, allowing growth to proceed [4]. Figure 3A shows that the S-acyl transferase null AKR1 C^500^S, presumably acting as a membrane localised set of AKR1 ankyrin repeats, is able to suppress STE4 induced cell cycle arrest, but AKR1 ΔN is not. Expression of the ankyrin repeats in a free soluble form does not suppress the effects of STE4 overexpression (Figure 3A). These data suggest that Gβγ interaction with AKR1 and suppression of Gβγ activity are dependent upon the ankyrin repeats and their membrane association through the transmembrane domains of AKR1 and not on the DHHC S-acyl transferase/PAT domain of AKR1.

One hypothesis is that the ankyrin repeats of AKR1 inhibit Gβγ by physically sequestering the Gβγ dimer. This would require a stable complex to be formed containing the ankyrin repeats of AKR1 and Gβγ Using the split-ubiquitin dual membrane yeast-2-hybrid system AKR1 and AKR1 C^500^S were shown to interact with STE18, the γ subunit of the βγ dimer (Figure 3B). This interaction is entirely dependent on the ankyrin repeats of AKR1; AKR1 ΔN does not interact with STE18 (Figure 3B). Importantly, the ankyrin repeats of AKR1 expressed on their own and anchored to the membrane interact just as effectively with STE18 as full length AKR1 (Figure 3C).

These results show that the ankyrin repeats of AKR1 can bind Gβγindependently of the rest of AKR1, providing that the ankyrin repeats are membrane associated, and AKR1 that is unable to catalyse S-acylation can still suppress Gβγ activity.

### The DHHC cysteine and ankyrin repeats of AKR1 are both required for full complementation of *akr1Δ* defects, but do not need to be on the same molecule

**Figure 4:**
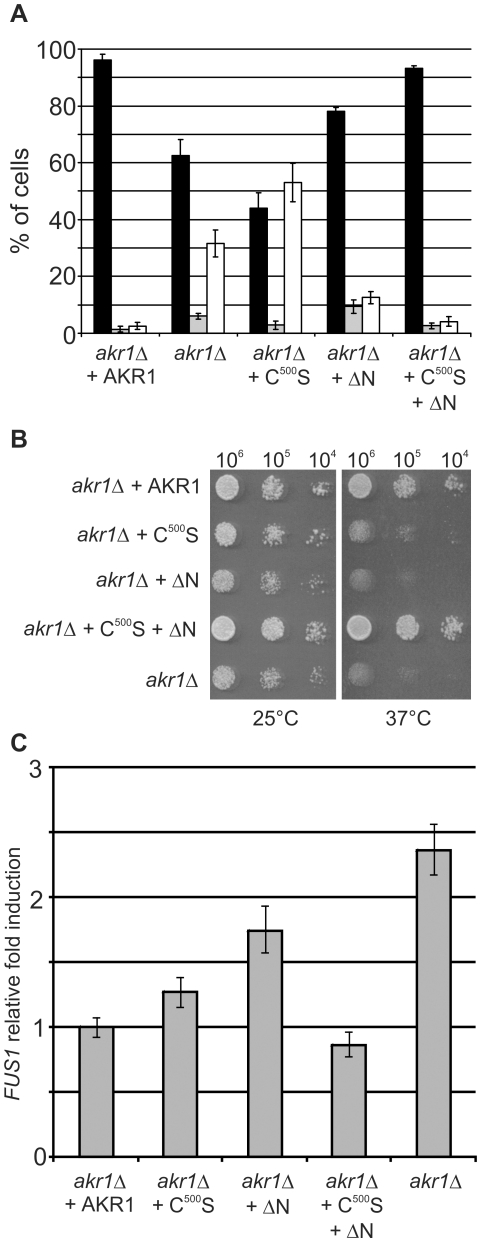
AKR1 requires both the DHHC domain and ankyrin repeats for rescue of phenotypic and mating pathway activation defects, but they do not need to be on the same molecule. (**A**) *akr1Δ* mutants show a reduction in WT morphology cells (black bars) and an increase in the proportion of cells with schmoo-like (grey bars) or multiple bud phenotypes (white bars). Introduction of AKR C^500^S to *akr1Δ* cells (*akr1Δ*+C^500^S) suppresses the schmooing phenotype but increases the number of cells showing a multiple bud phenotype. Introduction of AKR1 ΔN to *akr1Δ* cells (*akr1Δ*+ΔN) fails to suppress the schmooing phenotype but does reduce the number of cells that show a multiple bud phenotype. Co-expression of AKR1 C^500^S and AKR1 ΔN (*akr1Δ*+C^500^S+ΔN) in *akr1Δ* cells restores all morphological phenotypes to that observed for expression of full length WT AKR1 (*akr1Δ*+AKR1). The data represents the average of 3 independent experiments with >220 cells counted per genotype per experiment. Representative images of cultures used to generate these data are shown in Figure S3. Error bars represent 1 standard deviation. (**B**) *akr1Δ* mutants show a temperature sensitive phenotype with growth occurring at 25°C but not at 37°C. Introduction of AKR C^500^S (*akr1Δ+*C^500^S) or AKR1 ΔN to *akr1Δ* cells (*akr1Δ*+ΔN) fails to suppress the temperature sensitive phenotype. Co-expression of AKR1 C^500^S and AKR1 ΔN (*akr1Δ* C^500^S+ΔN) in *akr1Δ*cells restores temperature resistance as effectively as full length WT AKR1 (*akr1Δ* AKR1). (**C**) *akr1Δ*mutants show low level induction of the mating pathway in the absence of mating pheromone as measured by real time PCR of *FUS1* mRNA. Introduction of AKR1 C^500^S to *akr1Δ*cells suppresses *FUS1* induction significantly compared to *akr1Δ* while AKR1 ΔN only partly suppresses *FUS1* induction. Error bars represent a 95% confidence interval calculated from 3 technical replicates. Data are representative of 3 independent experiments.

The data presented above indicate that, although AKR1 dependent S-acylation relies upon DHHC function, the ankyrin repeats may serve to increase the efficiency of the S-acylation reaction by unknown means and can bind Gβγ independently of the DHHC domain. In light of these findings we dissected the roles and contribution of each domain, assessing the ability of AKR1 C^500^S and AKR1ΔN to complement the known phenotypes of *akr1Δ* mutants. Yeast cells lacking AKR1 are misshapen and frequently show abnormal growth including failure to separate after cytokinesis, elongated cells and branching at bud sites. This is believed to be due to simultaneous activation of both the vegetative and mating pathways due to poor suppression of basal Gβγ signalling and faulty YCK2 S-acylation [1], [3], [4]. During pilot experiments *akr1Δ* yeast expressing a version of AKR1 lacking both the ankyrin repeats and DHHC cysteine showed no difference in any of the phenotypes tested here compared to the *akr1Δ* mutant (data not shown) indicating that AKR1 does not contain any additional domains that affected our observations. *akr1Δ* yeast expressing AKR1 C^500^S do not show any restoration of the morphological phenotypes and actually have a higher proportion of cells that fail to separate after cytokinesis (multiple bud phenotype, Figure 4A, Figure S3). This indicates that YCK2 function is not restored but cell cycle progression is not impaired.

*akr1*Δ yeast expressing AKR1 ΔN show significant increases in the proportion of wild type morphology cells, a slight increase in cells with a schmoo like morphology and a decrease in the number of cells showing branching or pseudohyphal morphology compared to *akr1Δ* cells (Figure 4A, Figure S3). This indicates that YCK2 function is restored and that the majority of AKR1 function required to maintain correct cell shape is provided by the DHHC domain. However, mating pathway suppression has not occurred in these cells, which still have a higher tendency to schmoo and go into cell cycle arrest than cells expressing full-length AKR1. Co-expression of AKR1 C^500^S and AKR1 ΔN fully restores wild type morphology to *akr1Δ* cells (Figure 4A, Figure S3). These data demonstrate that while both domains are required to fully complement the growth and morphological defects observed in *akr1*Δ mutants the two domains do not need to be on the same molecule.

*akr1Δ* yeast cells display temperature sensitivity with mortality increasing with elevated temperatures [4], [5]. Yeast expressing either the S-acyl transferase null AKR1 C^500^S or the ankyrin-repeatless AKR1 ΔN do not show restoration of this phenotype. However, co-expression of AKR1 C^500^S and AKR1 ΔN restores temperature tolerance, again demonstrating that the DHHC domain and ankyrin repeats of AKR1 are not required to be on the same molecule for full restoration of *akr1Δ* phenotypes (Figure 4B).

Defects in the vegetative morphology of *akr1*Δ mutants are due in part to up regulation of mating pathway signalling in the absence of mating pheromone without full repression of vegetative pathways [1], [4]. In wild type cells perception of mating pheromone by STE3 leads to the activation of the STE20/STE11/STE7/FUS3 MAPK cascade through Gβγ ultimately switching yeast from vegetative to mating state, activating downstream genes such as *FUS1* which are required for mating, and inducing cell cycle arrest. In *akr1*Δ mutants basal Gβγ signalling is not suppressed and *FUS1* is constitutively expressed at low levels [1], [4]. AKR1 and AKR1 C^500^S are capable of suppressing basal *FUS1* transcription as measured by *FUS1* mRNA levels (Figure 4C) although AKR1 C^500^S does not repress FUS1 transcription quite as well as AKR1. This may be due to the reduced levels of AKR1 C^500^S in the cell compared to AKR1 described earlier (Figure 1C) or DHHC function being required for full suppression. *akr1*Δ mutants expressing AKR1 ΔN show levels of FUS1 activation that are greater than cells expressing AKR1 or AKR1 C^500^S but lower than in *akr1*Δ mutants, suggesting that the ankyrin repeats, rather than the DHHC domain, are the main suppressors of FUS1 transcription by AKR1. Co-expression of AKR1 ΔN and AKR1 C^500^S suppresses FUS1 to similar levels as full length AKR1. These data are supported by growth rate analysis that indicates that co-expression of AKR1 C^500^S and AKR1 ΔN in *akr1Δ*cells restores WT growth rates. AKR1 C^500^S expressed on its own in *akr1Δ*cells grows slightly better than *akr1Δ*, presumably because Gβγ induced cell cycle arrest is suppressed by the ankyrin repeats even though cell division and correct morphogenesis is still impaired. AKR1 ΔN expressing *akr1Δ*cultures grow more slowly than *akr1Δ* and do not reach the same levels of culture saturation (Figure S4). This is likely due to restoration of YCK2 and other S-acylation dependant functions resulting in largely normal morphological processes but with an inability to suppress Gβγ induced cell cycle arrest leading to a slow growth phenotype. All of these data combined indicate that suppression of the mating pathway in the absence of mating pheromone is achieved by both the ankyrin repeats and DHHC domain of AKR1 acting in concert by independent and distinct mechanisms within the same network.

## Discussion

AKR1 suppresses basal Gβγ-mediated mating pathway signalling in the absence of mating pheromone and promotes the maintenance of the vegetative state [1], [4]. We found that both the ankyrin repeats and DHHC domain of AKR1 contribute to this suppression. For many of the phenotypes we tested both the ankyrin repeat and DHHC domains are required, but they do not need to be on the same molecule indicating that AKR1 is a bi-functional protein with each function acting on different processes within the same pathway or network. AKR1 has previously been shown to be a PAT, and we show that part of the function of the ankyrin repeats is to increase S-acylation of YCK2 by an unknown mechanism. Interestingly the ankyrin repeats are not required on the same molecule as the DHHC PAT function to promote YCK2 S-acylation indicating that it may not be a direct effect. Our data suggest that the AKR1 ankyrin repeats continue to affect aspects of Gβγmediated mating pathway signalling even when there is no detectable AKR1 PAT activity in the cell (Figure 3, 4). We also show that AKR1 molecules are S-acylated at locations other than the DHHC cysteine, and that this increases the abundance of AKR1 in the cell. Our results prompt a reassessment of AKR1 and PAT research to date, especially recent attempts to study S-acylation enzymology and kinetics, and suggest several new lines of enquiry for future work on this and related proteins in other eukaryotic cells.

### AKR1 increases its own S-acylation at sites outside the DHHC motif

AKR1 molecules are able to increase each other's S-acylation at sites outside of the DHHC motif and this appears to promote the stability or reduce the turnover of AKR1 (Figure 1A, 1B, 1C). S-acylation of DHHC PATs outside of the DHHC cysteine has been reported for DHHC-5, -6 and -8, from humans in a proteomics study [22] but it is not know if their S-acylation occurs *in-cis* or *in-trans* either by themselves or other PATs. The motif identified as being S-acylated in these proteins does not exist in AKR1 or other yeast DHHC PATs indicating that the S-acylation event identified in our study occurs at a novel site. The simplest explanation for our data is that AKR1 molecules S-acylate each other directly using intermolecular rather than intramolecular transfer of S-acyl groups. This conclusion is supported by evidence that molecules of AKR1 with an intact DHHC domain cysteine are an absolute requirement for AKR1 S-acylation to occur outside of the DHHC domain (Figure 1A, 1B). However, stable interaction between AKR1 monomers is not detectable by co-immunoprecipitation [12] or yeast-2-hybrid (Figure S2) assays and the possibility that AKR1 monomers somehow target each other for S-acylation by other S-acyl transferases cannot be completely ruled out, although interpretation of *in-vitro* data on auto-S-acylation of the DHHC PAT ERF2 [18] in light of our findings suggests that this is unlikely.

The ability to purify an S-acylated form of a PAT has been used to propose that they form a stable intermediate with a covalently bound S-acyl group before transferring the S-acyl group to the substrate and that the DHHC cysteine is likely to be the catalytic site [5], [13], [16], [17], [18], [19], [23]. These assays have been largely interpreted assuming that S-acylation only occurs on the DHHC cysteine. Our discovery that AKR1 is S-acylated at sites other than the DHHC cysteine potentially casts doubts on some of the conclusions drawn from these assays. This additional S-acylation could mask the binding of S-acyl groups to the active site and transferral to substrates and provides a possible alternative S-acylation substrate for the PAT that could interfere with attempts to investigate the kinetics or mechanism of the S-acylation reaction.

These ideas may offer a solution to an unexplained phenomenon observed in a recent study examining the kinetics of S-acylation of RAS2 by ERF2, another yeast DHHC PAT [18]. ERF2 auto-S-acylates in the presence of palmitoyl-CoA in two distinct stages; a rapid initial phase, lasting only a few seconds at room temperature, followed by a prolonged steady state phase. The steady state phase is a cycle of auto-S-acylation of ERF2, releasing free CoASH as a result of ERF2 covalently binding palmitate, followed by hydrolytic turnover of the palmitoyl group to release free palmitate. This cycle proceeds at a steady rate; in the presence of excess palmitoyl-CoA, CoASH release and palmitate hydrolysis occurs in a 1∶1 ratio indicating that for every palmitate hydrolysed a new one is bound. This cycle cannot however explain the initial rapid phase where CoASH is released, indicating S-acylation has occurred, but palmitate is not hydrolysed from ERF2 (no turnover). Palmitate hydrolysis does not follow the same initial rapid kinetics as CoASH release but proceeds at the same rate as the later stage steady state CoASH release throughout [18], indicating that much of the initial palmitate bound by ERF2 is not hydrolysed and turned over. These intriguing kinetic data are commented on by the authors [24] but not fully explained. Our results suggest a plausible explanation where the rapid initial CoASH release and increase in S-acylated ERF2 and lack of palmitate hydrolysis are due not only to DHHC domain auto-S-acylation, but also to stable addition of palmitate to ERF2 by other ERF2 molecules at a site other than the DHHC cysteine, in a manner consistent with our results reported here with AKR1. This extension of the previous hypothesis could be tested initially by determining whether ERF2 carrying the C^203^S mutation is S-acylated in WT but not in *erf2Δ* cells. This will give an indication as to whether ERF2 influences its own S-acylation at non-DHHC sites. If this proves to be the case then identification and mutation of the site followed by repeating the “auto-S-acylation” kinetic analysis of ERF2 and ERF2 lacking the non-DHHC S-acylation site would allow comparison of kinetics and testing as to whether S-acylation *in trans* of ERF2 affects or obscures the true kinetics of WT ERF2. These data may then help explain the intriguing results reported previously [24].

Our observations should not greatly affect the data obtained from the steady state kinetics observed in these studies but may complicate observations at early time points. Measurements using CoASH release would include that released during the S-acylation of ERF2 at the non-DHHC site and therefore lead to an overestimation of the initial rate at the DHHC site. They also indicate that over estimation of the steady state levels of binding of palmitoyl-CoA is likely as the covalently bound non-transferable palmitoyl-CoA added by ERF2 to other ERF2 molecules during the initial stages of the reaction would be included in the count. These data indicate the need for careful consideration of this new factor in experimental set up and design. The use of thioester cleaving reducing agents that will liberate free S-acylatable sulfhydryls during purification of the PAT may need to be limited or taken into consideration during assays. We suggest that reactions to measure kinetics should be initiated by the addition of substrate rather than palmitoyl-CoA or that PAT that has been pre-treated with cold palmitoyl-CoA to eliminate signal from non-catalytic S-acylation sites should be used.

The S-acylation of AKR1 and ERF2 outside of the DHHC cysteine might restrict their *in-vivo* sub-cellular and membrane microdomain distribution, as has been described for other S-acylated proteins [25], [26], [27], [28]. If so then abnormally S-acylated AKR1 could have an undefined or aberrant distribution within cellular membrane compartments and membrane microdomains causing a decrease in interaction efficiency with partner proteins such as Gβγ or S-acylation targets resident in specific membrane microdomains. Alternatively, S-acylation of AKR1 could reduce its degradation and turnover, as has been proposed for TLG1 [29].

The data presented here are also compatible with the idea that each S-acylation reaction, such as YCK2 S-acylation by AKR1, requires 2 sequential S-acyl transferase reactions, once on AKR1 itself and finally on YCK2. Our data indicate that S-acyl groups are transferred from AKR1 to a non-DHHC cysteine of AKR1 *in-trans* in a DHHC domain dependant manner. This version of AKR1, S-acylated at the non-DHHC site, could be the active transferase for substrate S-acylation. It is still not clear whether the DHHC cysteine is actually the active site.

### The ankyrin repeats of AKR1 contribute to the regulation of Gβγ signalling by two mechanisms

*akr1Δ* cells show reduced constitutive ligand independent STE3 endocytosis [2], and defective YCK2 S-acylation [5] resulting in increased basal mating pathway induction through Gβγ [1], [4]. *akr1Δ* cells also show defective cytokinesis resulting in multiple budding events that frequently fail to separate [1] leading to multi-nucleate cell masses [4]. Interestingly *akr1Δ* mutants do not show defects in mating pathway induction after treatment with mating pheromones indicating that AKR1 acts to suppress mating pathway activation but only in the absence of pheromone [1], [4].

We show that the ankyrin repeats of AKR1 contribute to AKR1 function in at least two ways. Firstly, they promote the S-acylation of YCK2 by an unknown mechanism (Figure 2) that does not require the ankyrin repeats and DHHC domain to be on the same molecule. These data, coupled with data indicating that AKR1 does not interact with itself, suggest that the influence of the ankyrin repeats on YCK2 S-acylation is likely to be indirect, rather than directly affecting the S-acylation reaction itself. Secondly, we show that the ankyrin repeats have strong effects on Gβγ signalling in the absence of detectable AKR1 S-acylation activity. Data in Figures 1 A and B and Figure 2 confirm previously published results that AKR1 C^500^S has no detectable S-acylation activity [5] and that it essentially functions as a membrane localised form of the AKR1 ankyrin repeats, yet we show that reintroduction of this protein into *akr1Δ* cells suppresses the majority of *FUS1* up regulation observed in *akr1Δ* cells as a result of mating pathway activation (Figure 4C). AKR1 C^500^S can also counter the effects of STE4 over expression to permit growth (Figure 3A) and partially rescue the temperature-sensitivity of *akr1Δ* cells (Figure 4B). Furthermore, AKR1 ΔN cannot suppress STE4 over expression induced cell cycle arrest (Figure 3A), schmooing (Figure 4A), or the temperature-sensitivity of *akr1Δ* (Figure 4B). We also demonstrate that the ankyrin repeats on their own, when artificially anchored to the membrane, interact with STE18 in yeast-2-hybrid assays (Figure 3B, C). Interestingly the ankyrin repeats, when expressed in a soluble form, do not suppress the effects of STE4 over expression (Figure 3A). Taken together, these results suggest that the ankyrin repeats of AKR1 play a significant role in the regulation of Gβγactivity that is independent of the S-acylation activity of AKR1. These data also indicate that the ankyrin repeats require membrane association, either through the transmembrane domains of AKR1 or by artificial recruitment in the DUALmembrane yeast-2-hybrid system, to have these effects. This is perhaps unsurprising given that Gβγ is also membrane associated through prenylation and S-acylation of STE18/Gγ [20].

### Overall contribution of AKR1 to the suppression of Gβγ induced mating pathway activation in the absence of pheromone

The interpretation of literature to date suggests that AKR1 is a PAT [5] and that it somehow suppresses Gβγ induced mating pathway activation [1], [2], [4], presumably via its PAT activity and possibly through YCK1 and 2. Here we present evidence that this view should be revised to incorporate ankyrin repeat function that does not require the AKR1 DHHC domain. Given that both the ankyrin repeats and DHHC domain affect the same overall processes and pathways it is perhaps not surprising that each is able to either fully or partially substitute for AKR1 but one is usually more effective than the other (Figures 1, 2, 3 and 4). Importantly, co-expression of AKR1 C^500^S and AKR1 ΔN restores all of the phenotypes tested even though the ankyrin repeats and S-acylation domain are on different molecules (Figure 4A, 4B, 4C). This indicates that although direct interaction of AKR1 monomers has not been shown to occur ([12], Figure S2) the functions of each domain are both required at the same time to adequately suppress the basal activation of the mating pathway.

**Figure 5:**
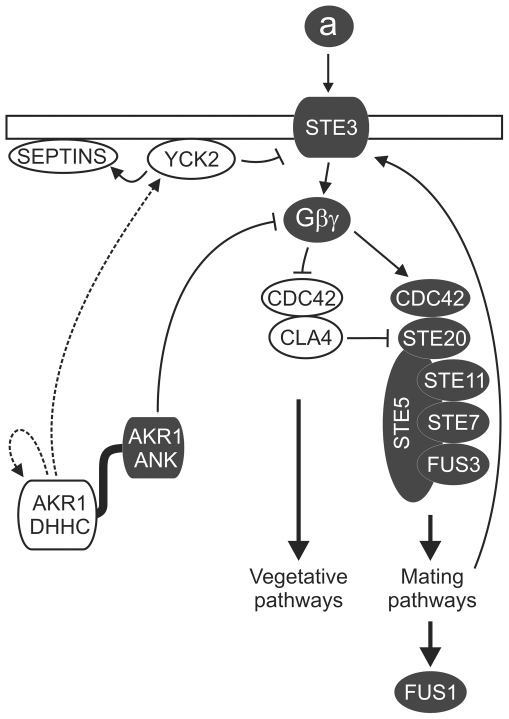
Speculative model of AKR1 function suggested by our results and the literature. Our results are consistent with a model where the ankyrin repeats suppress aspects of Gβγ-induced mating pathway activation independently of the DHHC domain. In the absence of mating pheromone the ankyrin repeats suppress basal Gβγ signalling through the STE20 MAPK pathway, possibly by directly binding (see results in Figure 3) and sequestering Gβγ or a Gβγ containing complex released by basal STE3 activity away from downstream mating pathway components The DHHC domain suppresses inappropriate mating pathway activation by ensuring STE3 levels are kept low via the S-acylation of YCK2, which in turn would phosphorylate STE3 at the plasma membrane leading to STE3 endocytosis. These processes would both help to maintain low levels of basal Gβγ signalling and promote vegetative growth. This model suggests the following explanations for the phenotypes that we have observed. The low level mating pathway activation in AKR1ΔN expressing cells could be due to free Gβγ released by basal STE3 activity partially activating the mating pathway in the absence of AKR1 ankyrin repeats (which would otherwise suppress Gβγ activity). AKR1 C^500^S, unable to S-acylate YCK2, would lead to a failure in constitutive endocytosis of STE3 [2], [3], and the resultant elevated levels of STE3 would lead to elevated basal Gβγ release [2], exceeding the buffering potential of the ankyrin repeats. This would explain the slightly poorer performance of AKR1 C^500^S in suppressing *FUS1* induction (Figure 4C). Domains of AKR1 and proteins with roles predominantly in vegetative growth are represented in white while those with roles predominantly in mating are represented in grey. Broken lines represent S-acylation activity of AKR1. Arrowed lines represent positive effects on function, barred lines represent negative effects on function. AKR1 DHHC – DHHC domain of AKR1, AKR1 ANK – ankyrin repeats of AKR1.

YCK2 coordinates growth, septin formation, schmoo polarization and cell division during the cell cycle. To correctly perform these functions YCK2 cycles between different subcellular locations depending on the stage of the cell cycle [30] and requires S-acylation by AKR1 for this to occur [5], [10]. Our data support the previous hypotheses that the gross phenotypes observed in *akr1Δ* mutants, which are superficially similar to *yck1Δ yck2Δ* double mutants [9], are the result of simultaneous vegetative and mating pathway activation and an inability to coordinate correct cell division and organise cell polarity [1], [4]. We propose that in *akr1Δ* cells YCK2 and other S-acylated targets of AKR1 are functionally impaired as they are not S-acylated and therefore unable to coordinate the functions required for correct cellular function and basal Gβγ signalling is not suppressed by the AKR1 ankyrin repeats and partial cell cycle arrest occurs. These defects in the two different biochemical functions of AKR1 allow both mating and vegetative pathways to be concurrently, but incorrectly, active causing the gross phenotype of *akr1Δ* cells. Analysis of growth rates indicates that AKR1 ΔN actually grows more slowly (Figure S4) and shows a higher rate of schmoo formation (Figure 4A) than *akr1Δ*. This is likely due to restoration of YCK2 function (e.g. Figure 2). We speculate that restoration of YCK2 function in AKR1 ΔN cells induces cell cycle arrest to a greater degree than in *akr1Δ* as the cell is able to correctly respond to vegetative and mating morphogenic signalling, such as controlling the correct number of budding/schmoo events and co-ordinating cell division, but without the ability provided by the ankyrin repeats to suppress basal Gβγsignalling,resulting in cell cycle arrest. A speculative model that can account for our new data on AKR1 function whilst placing it in the context of earlier work [1], [2], [4], [5] is shown in Figure 5.

### Potential function of AKR1-like proteins

Given our data the origin of AKR1 like proteins may be a result of the need for close temporal coordination between two overlapping functions acting within the same overall network. AKR1 like proteins containing both DHHC domains and ankyrin repeats have been identified in eukaryotes from all three kingdoms [1], [4], [13], [31], [32]. This indicates that the origin of DHHC domain proteins containing ankyrin repeats is ancient and may have conserved purpose. This is supported by evidence that both human HIP14 [31] and *Arabidopsis* TIP1 [13] homologues of AKR1 are able to restore defects observed in *akr1Δ* mutants that are associated with loss of both ankyrin repeat and PAT function. Both HIP14 and TIP1 are able to act as S-acyl transferases [13], [14], [15] but these data also suggest that the fundamental roles of the ankyrin repeats have also been conserved in these proteins.

A similar situation to that observed in yeast, where AKR1 acts to suppress mating pathway activation by the STE20 MAPKKKK in favour of vegetative growth pathway activation by the CLA4 MAPKKKK (Figure 5), occurs in mammals where the two opposing JNK and p38 MAPK pathways are regulated and switched by Gβγ. Over expression of the human AKR1 homologue HIP14 is able to increase activation of the mammalian JNK MAPK pathway and the ankyrin repeats are required for this [32]. UV stress or over expression of the appropriate UV responsive human Gβγ complex Gβ_1_γ_2_ in COS-1 cells activates the p38 MAPK cascade which directly suppresses JNK activation [33]. Sequestration of Gβ_1_γ_2_ by over expression of HIP14 could conceivably lead to increased activation of the JNK pathway by preventing the activation of the p38 MAPK pathway by Gβ_1_γ_2_. Available data are consistent with a model where the ankyrin repeats of AKR1 like proteins sequester Gβγ dimers released from unstimulated receptors (i.e. basal or leaky signalling in the receptor off state) or from recycling of signalling components after signalling events. This model would essentially put the ankyrin repeats of AKR1-like proteins as threshold or gating switches that suppress basal Gβγ signalling in the absence of receptor stimulation. After activation of GPCRs the level of Gβγ released from activated Gαβγ heterotrimers in the cell would exceed AKR1-like proteins' buffering ability and the downstream signalling pathway would be activated.

The human AKR1 homologue HIP14 is responsible for the majority of Huntingtin (Htt) S-acylation activity in mammalian cells [15]. Recent data indicates that addition of the ankyrin repeats of HIP14 to another PAT, DHHC-3, in neuronal cells increases the efficiency of Htt S-acylation by over expressed DHHC-3 [34]. PATs have low substrate specificity and are promiscuous enzymes, especially when over expressed [35], [36], and a recent report suggests that mammalian PAT specificity for peripheral membrane proteins, such as Htt, is minimal or entirely absent [36]. DHHC-3 is reported to have particularly wide substrate specificity and is capable of S-acylating Htt even in the absence of prosthetic HIP14 ankyrin repeats [34]. Whether the observed increase in S-acylation is a result of direct binding of Htt by the ankyrin repeats followed by S-acylation or altered localisation or stability of the fusion has not been addressed. The observed data may also be explained by the ankyrin repeats recruiting the low specificity S-acylation activity of over expressed DHHC-3 to a usually HIP14 containing Htt S-acylation complex.

It will be interesting to discover whether AKR1-like proteins in other eukaryotes have multiple functions that act within a single overall network, as appears to be the case for AKR1 in the yeast mating pathway. Studies in other eukaryotes would help elucidate whether the AKR1 state of having both functions act within the same pathway is derived or ancestral.

## Materials and Methods

### Strains and plasmids

6xHIS/3xHA/FLAG epitope tagged versions of AKR1 expressed from its native promoter (pRS316, pRS315 [37] and pESC 2μorigin based) were created by PCR based recombination in yeast using pND1434 [5] as the base AKR1 template. AKR1 C^500^S has been described previously [5]. AKR1 ΔN carries a deletion of amino acids 3–302 inclusive. Expression of free AKR1 ankyrin repeats involved deletion of amino acids 303–755 inclusive. Recombinant plasmids were screened by colony PCR and sequenced before use. Over expression of *STE4* was achieved by cloning *STE4* downstream of the *GAL1* promoter of pJB9. Expression of GFP-YCK2 from the GAL1 promoter was achieved using pJB1 [30]. Vectors for yeast-2-hybrid were produced by cloning AKR1 variants lacking start and stop codons in frame into the SfiI sites of pBT3-STE. AKR1 ankyrin repeats (covering amino acids 2–311) for yeast-2-hybrid were cloned into the Sfi1 sites of pBT3-OST4. pBT3-OST4 was created by replacing the STE2 leader peptide of pBT3-STE with the entire OST4 ORF lacking start and stop codons by homologous recombination in yeast. STE18 was cloned, lacking the start codon, in frame with the SfiI sites of pPR3-N.

All yeast strains used for characterising AKR1 cellular functions were isogenic with BY-series strains used for the EUROscarf deletion program [38] WT BY4742 genotype (accession number - Y10000) - *MATα; his3Δ1; leu2Δ0; lys2Δ0; ura3Δ0*. *akr1Δ* genotype (accession number - Y13623) - *MATα; his3Δ1; leu2Δ0; lys2Δ0; ura3Δ0; YDR264c::KanMX4*. Yeast-2-Hybrid analysis was performed in strain NMY51 (*MATa his3Δ200 trp1-901 leu2-3,112 LYS2::(lexAop)4-HIS3 ura3::(lexAop)8-lacZ (lexAop)8-ADE2 GAL4*) obtained from DUALsystems biotech. Yeast transformation was performed as described [39]. Yeast were cultured using standard laboratory techniques and media.

### Growth assays

For temperature sensitivity assays strains were grown to mid log phase (O.D._600_ 0.6–0.8) in SD-LU, and serial dilutions from 10^6^ to 10^4^ cells per ml prepared in SD-LU and 10 µl aliquots plated onto SD-LU plates. Plates were incubated at 25°C and 37°C for 48–60 hours. Strains for suppressing STE4 induced growth arrest were grown to mid-log phase in SD-LU, washed once in distilled water and resuspended in 0.9% NaCl. Serial dilutions from 10^6^ to 10^4^ cells per ml were prepared and 10 µl aliquots spotted onto YPD and YPGalRaf plates and incubated at 25°C for 36 hours (YPD) or 48 Hours (YPGalRaf).

### S-acylation assay

S-acylation assays using a modification of the biotin switch method [40] were performed as described [41]. Briefly, samples were incubated with N-ethylmaleimide to block free sulfhydryls. Samples were then incubated with biotin-HPDP (Thermo Scientific) in the presence or absence of the thioester cleaving reagent hydroxylamine. Samples were purified using Neutravidin resin and eluted into 2× SDS sample buffer. Protein S-acylation state was determined using antibodies against the protein of interest as a function of recovery by the Neutravidin beads. Induction of YCK2:GFP for use in S-acylation assays was performed by adding galactose to mid-log phase cultures in selective synthetic raffinose medium. After 90 minutes of induction, glucose was added to 2% and cultures incubated for a further 90 minutes before harvesting.

### Western blotting

Protein extracts were prepared from yeast cells using standard methods [42] or as described for S-acylation assays [41]. Western blots to detect epitope tagged AKR1 variants were performed using anti-HA polyclonal antibody (Santa Cruz Biotechnologies, sc-805) diluted 1∶200 in 5% skimmed milk powder, 0.05% Tween-20, TBS (5%MTBST). YCK2-GFP was detected using anti-GFP polyclonal antibody (Santa Cruz Biotechnologies, sc-8334) diluted 1∶200 in 5%MTBST. Primary antibodies were detected using goat anti-rabbit IgG-HRP conjugate (Santa Cruz Biotechnologies, sc-2004) diluted 1∶5000 in 5%MTBST. Blots were exposed to Hyperfilm ECL (GE Healthcare) using ECL advance reagents (GE Healthcare).

### Yeast-2-hybrid

Interaction analysis was performed using the Dual membrane yeast-2-hybrid assay (Dualsystems Biotech). To assay interactions yeast were grown to mid log phase, collected by centrifugation, washed once in distilled water and resuspended in 0.9% NaCl. Serial dilutions from 10^6^ to 10^4^ cells per ml were prepared and 10 µl aliquots were spotted onto synthetic dextrose media lacking the appropriate amino acids for plasmid selection (SD-LW, control) and for reporter gene expression (SD-LWHA+1 mM 3-aminotriazole, experimental) and incubated at 25°C until strong growth was observed (48–60 hours).

### Real time PCR

Total cellular DNA free RNA was extracted using a RNeasy mini kit with on column DNAseI digestion (Qiagen) from mid-log phase yeast cells grown in selective medium. *FUS1* expression levels were standardised to *IPP1* expression as *IPP1* expression is extremely stable over a wide range of conditions [43]. 2 µg of RNA was reverse transcribed using a High Capacity Reverse Transcriptase kit with random hexamers (Applied Biosystems). Quantitative real time PCR was performed on a 7300 Real Time PCR system (Applied Biosystems) using FastStart Universal SYBR Green Master Mix with ROX as a passive control (Roche). PCR products were validated using melt curve analysis. Primers used were FUS1f - GGTGTGATATTGTCATCAAGTTGCA, FUS1r - TGATGTTGGTAACGGCACATG, IPP1f - CAAGGGTATTGATTTGACCAATGTT, IPP1r - GAGGCAGCCTTGGAGTAGGTT.

## Supporting Information

Figure S1 **Cartoon structure of AKR1 and AKR1 variants used in this study.** ARPS – 6 ankyrin repeats, DHYC – S-acyl transferase domain, DHYS – C^500^S mutant version of the AKR1 DHHC domain unable to act as a PAT. Solid black boxes represent transmembrane spans.
(TIF)

Figure S2 **A.** AKR1 variants do not interact with each other in pairwise interaction assays. AKR1 variants in pBT3-STE (bait) were screened for interaction with AKR1 variants in pBT3-STE, pAI positive control vector (+), pDL2 negative control vector (−) and empty prey vector (pPR3-STE). All strains grew on media selective for plasmids (-LW) but only pAI interaction with AKR1 variants supported growth on selective media (-LWHA) indicating that the bait constructs are functional. **B.** AKR1 variant prey constructs are expressed in AKR1 variant bait backgrounds. A – pPR3-STE AKR1, C - pPR3-STE AKR1 C^500^S, N - pPR3-STE AKR1 ΔN. Bait constructs are indicated at the bottom of the figure. AKR1 and AKR1 C^500^S are indicated by the upper arrow and AKR1 ΔN by the lower arrow.
(TIF)

Figure S3 **Representative images of** ***akr1Δ*** **cultures expressing AKR1, AKR1 C^500^S, AKR1 ΔN and AKR1 C^500^S+AKR1 ΔN used to generate data for** **Figure 1A** **.** Scale bar represents 10 µm. *akr1Δ* cells are large, highly branched, and multinucleate. *akr1Δ* cells co-expressing AKR1 or AKR1 C^500^S+AKR1 ΔN have wild type phenotypes. *akr1Δ* cells expressing AKR1 C^500^S alone are less likely to schmoo, but still produce multiple buds. *akr1Δ* cells expressing AKR1 ΔN alone have fewer multiple buds than *akr1Δ* cells but schmoo more than wild type.
(TIF)

Figure S4 **Typical growth curves for the strains used in this study.** Cultures were grown in synthetic dropout media (SD-LU) at 25°C with shaking to an OD_600_ of 0.8–1.2 and inoculated into SD-LU to an OD_600_ of 0.15. Cultures were grown at 25°C with shaking and monitored for 36 hours with OD_600_ measurements taken every hour. All strains expressing AKR1 variants are in the *akr1Δ* background.
(TIF)
