# Supplementary figures and images for: The Ankyrin Repeats and DHHC S-acyl Transferase Domain of AKR1 Act Independently to Regulate Switching from Vegetative to Mating States in Yeast
Source: PLoS One. 2011 Dec 8;6(12):e28799. doi: 10.1371/journal.pone.0028799 (PMC3234281; doi:10.1371/journal.pone.0028799)

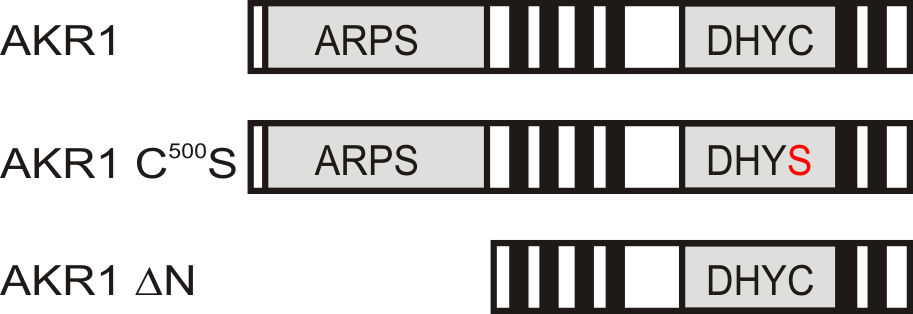

Supplement: Figure S1 — Cartoon structure of AKR1 and AKR1 variants used in this study. ARPS – 6 ankyrin repeats, DHYC – S-acyl transferase domain, DHYS – C500S mutant version of the AKR1 DHHC domain unable to act as a PAT. Solid black boxes represent transmembrane spans. (TIF) [file pone.0028799.s001.tif]

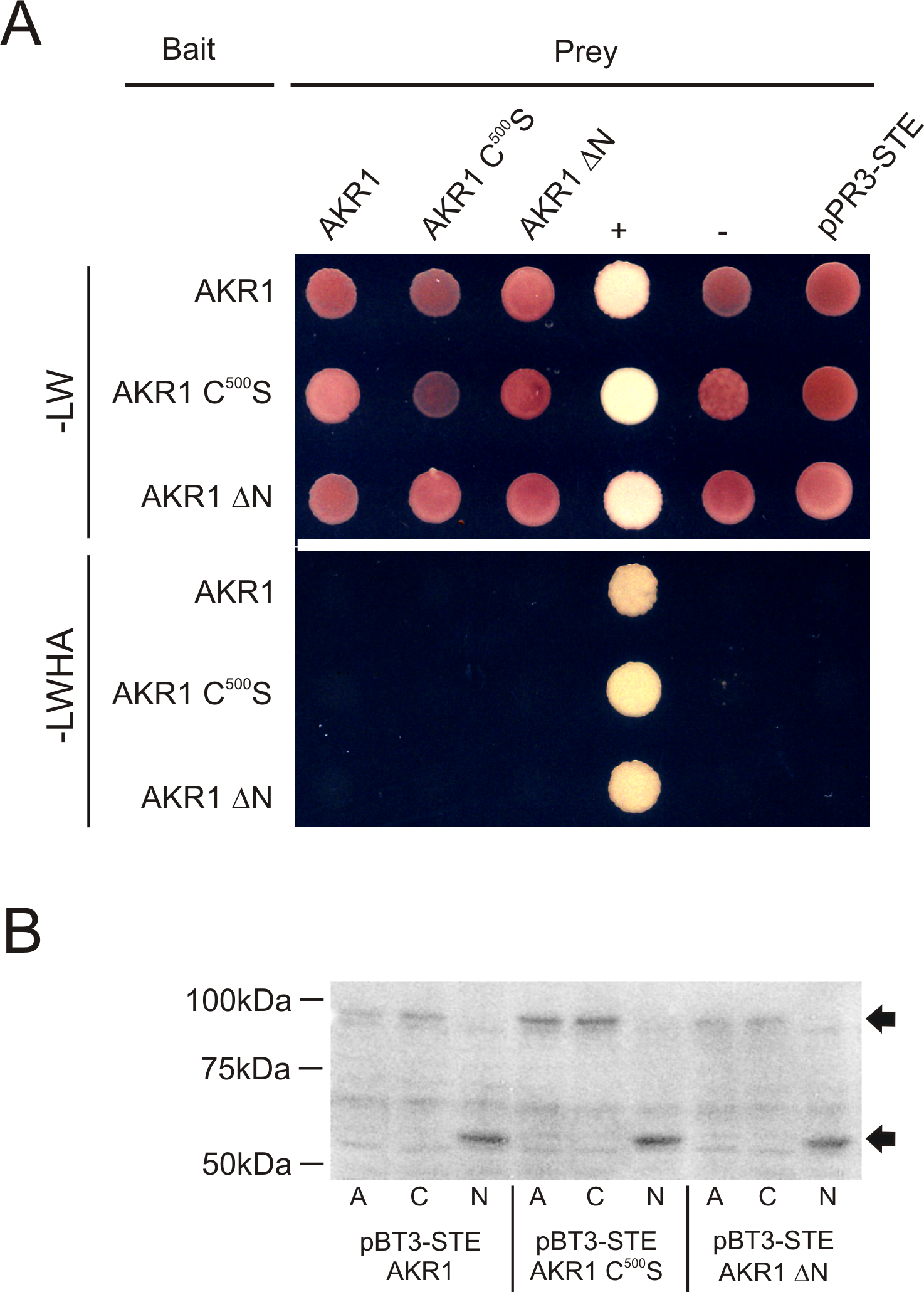

Supplement: Figure S2 — A. AKR1 variants do not interact with each other in pairwise interaction assays. AKR1 variants in pBT3-STE (bait) were screened for interaction with AKR1 variants in pBT3-STE, pAI positive control vector (+), pDL2 negative control vector (−) and empty prey vector (pPR3-STE). All strains grew on media selective for plasmids (-LW) but only pAI interaction with AKR1 variants supported growth on selective media (-LWHA) indicating that the bait constructs are functional. B. AKR1 variant prey constructs are expressed in AKR1 variant bait backgrounds. A – pPR3-STE AKR1, C - pPR3-STE AKR1 C500S, N - pPR3-STE AKR1 ΔN. Bait constructs are indicated at the bottom of the figure. AKR1 and AKR1 C500S are indicated by the upper arrow and AKR1 ΔN by the lower arrow. (TIF) [file pone.0028799.s002.tif]

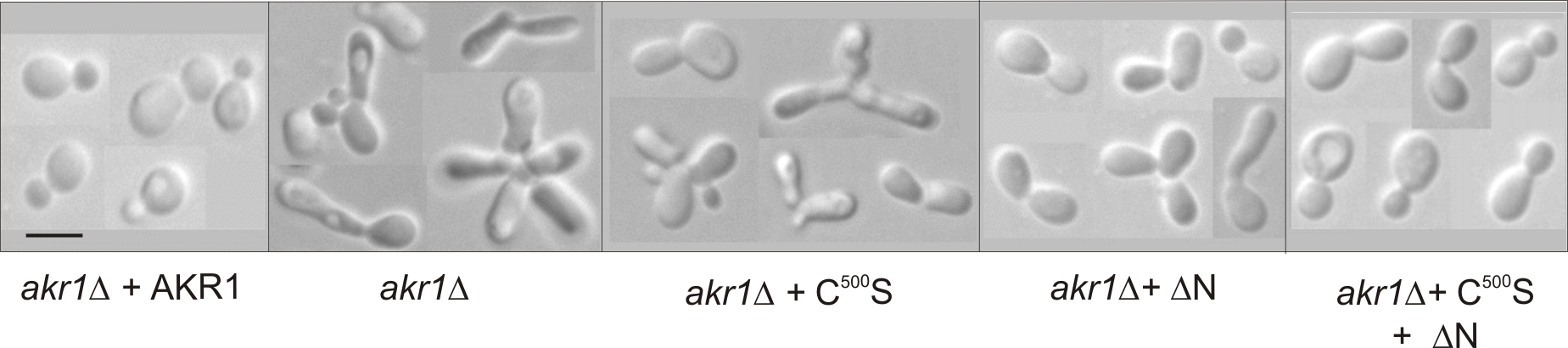

Supplement: Figure S3 — Representative images of akr1Δ cultures expressing AKR1, AKR1 C500S, AKR1 ΔN and AKR1 C500S+AKR1 ΔN used to generate data for Figure 1A . Scale bar represents 10 µm. akr1Δ cells are large, highly branched, and multinucleate. akr1Δ cells co-expressing AKR1 or AKR1 C500S+AKR1 ΔN have wild type phenotypes. akr1Δ cells expressing AKR1 C500S alone are less likely to schmoo, but still produce multiple buds. akr1Δ cells expressing AKR1 ΔN alone have fewer multiple buds than akr1Δ cells but schmoo more than wild type. (TIF) [file pone.0028799.s003.tif]

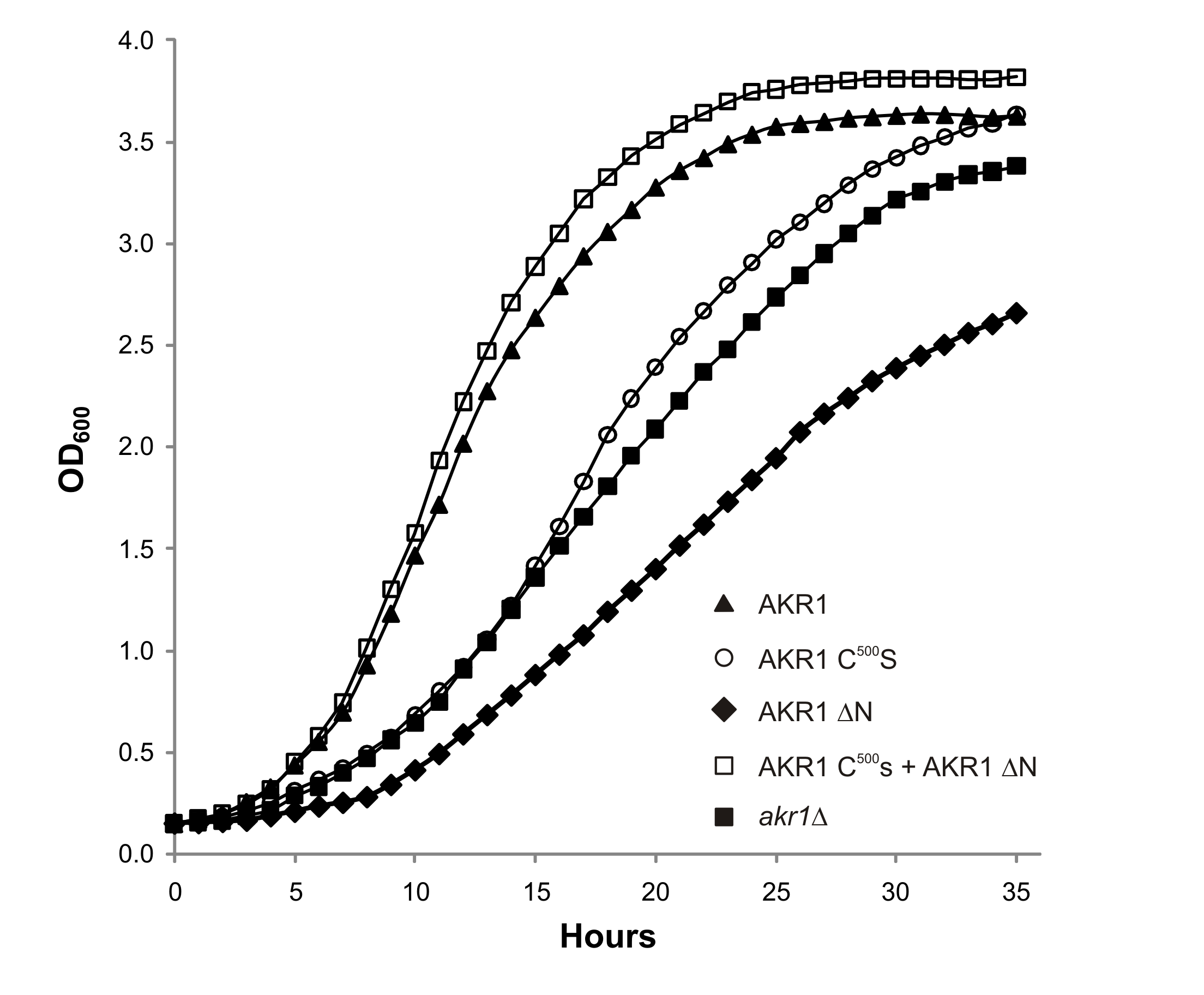

Supplement: Figure S4 — Typical growth curves for the strains used in this study. Cultures were grown in synthetic dropout media (SD-LU) at 25°C with shaking to an OD600 of 0.8–1.2 and inoculated into SD-LU to an OD600 of 0.15. Cultures were grown at 25°C with shaking and monitored for 36 hours with OD600 measurements taken every hour. All strains expressing AKR1 variants are in the akr1Δ background. (TIF) [file pone.0028799.s004.tif]
